# Expert consensus on the clinical application of enhanced external counterpulsation in elderly people (2019)

**DOI:** 10.1002/agm2.12097

**Published:** 2020-03-03

**Authors:** Shen Lin, Wang Xiao‐ming, Wu Gui‐fu

**Affiliations:** ^1^ Department of Geriatrics Qilu Hospital of Shandong Univeristy, Key Laboratory of Cardiovascular Disease Proteomics of Shandong Province Ji‐nan city China; ^2^ Department of Geriatrics Clinical Research Center for Geriatric Diseases Xi Jing Hospital of Air Force Medical University Xi‐an city China; ^3^ Department of Cardiovascular Medicine Research Center for Assisted Circulation Innovative Engineering Technologies The Eighth Affiliated Hospital of Sun Yat‐sen University Shen‐zhen city China

**Keywords:** enhanced external counterpulsation

## Abstract

Enhanced external counterpulsation (EECP) is a non‐invasive assisted circulation technique and a rich pool of evidence has accumulated for its clinical application in the prevention and management of multiple comorbidities in the elderly population, including angina, heart failure, ischemic cerebrovascular diseases, neurodegenerative diseases, sleep disorder, diabetes and its complications, ischemic eye diseases, sudden hearing loss and erectile dysfunction, as well as various psychological and psychiatric conditions. When applying EECP to elderly patients, emphasis should be placed on issues such as safety assessment, risk management and protocol individualization, as well as the monitoring of efficacy during and after treatment.

## 1 INTRODUCTION

Enhanced external counterpulsation (EECP) is a non‐invasive assisted circulation technique, which employs electrocardiogram‐gated sequential inflation of cuffs wrapped around calves, thighs and buttocks, which inflate and deflate during the cardiac cycle. It improves organ ischemia through a series of protective mechanisms. EECP was first introduced in the treatment of angina and was subsequently applied in various conditions including heart failure, ischemic cerebrovascular diseases and so on, with documented evidence of benefits.^1^, ^2^, ^3^, ^4^ Improvements in diabetes, ischemic eye diseases, sudden hearing loss, male erectile dysfunction, sleep disorders, etc have also been reported.^5^, ^6^, ^7^, ^8^, ^9^, ^10^ As a safe, non‐invasive and effective treatment, EECP has attracted interest from geriatricians who care for elderly with multiple comorbidities. Recent years have seen an ever‐expanding pool of evidence concerning the positive effects of EECP. In light of this, the Cardiovascular Group of the Geriatric Branch of the Chinese Medical Association, the Editorial Board of the *Chinese Journal of Geriatrics* and the Geriatric Group of the External Counterpulsation Branch of the Chinese Society of Biomedical Engineering have jointly produced this “Expert consensus on the clinical application of EECP in elderly people”, in order to standardize and promote the use of EECP in a geriatric context in China.

## 2 WORKING PRINCIPLES AND MECHANISM OF BENEFIT OF EECP

### 2.1 Working principles of EECP

EECP and the intra‐aortic balloon pump (IABP) are assisted circulation techniques. IABP as an invasive treatment is used for circulatory support in cardiogenic shock. As for the working principles, EECP and IABP are similar in that both achieve improvement in coronary circulation and myocardial contractility through electrocardiogram‐gated, mechanical diastolic augmentation in the aorta, but EECP is different from IABP in that EECP, by squeezing blood from the lower extremities, increases venous return, which in turns increases cardiac output. EECP has been proven to effectively improve perfusion to vital organs such as heart, brain and kidneys.^11^ During treatment, cuff inflation and deflation are precisely timed in accord with the opening and closing of the aortic valve through surface electrocardiogram. The proximal‐to‐distal manner of inflation/deflation cycle of calves, thighs and buttocks ensures that the proximal arteries are compressed later relative to the distal arteries, thus driving more arterial flow back to the aorta, enhancing diastolic augmentation.^12^ Sequential inflation/deflation is also more effective than non‐sequential protocol. The combination of lower extremities plus buttocks (termed EECP) is superior both in efficacy and comfort compared with the combination of lower extremities plus upper extremities.

### 2.2 Mechanism of benefit of EECP

#### 2.2.1 Effects related to immediate hemodynamics

Early studies of EECP focused on its hemodynamic effects. (1) Arterial pressure: flow pulsatility is unique to EECP hemodynamics, in which diastolic aortic pressure is increased by 26%‐157%.^13^ Its effects on systolic aortic pressure vary in different studies, which report a lowering of systolic pressure by 9‐16 mm Hg (1 mm Hg = 0.133 kPa). (2) Ventricular function: EECP increases cardiac output by an average of 25%.^14^, ^15^ (3) Coronary flow: EECP increases pressure by 16% and flow velocity by 109% on average in the coronaries.^14^

#### 2.2.2 Effects related to vascular biology

As our understanding of vascular biology deepens, the molecular mechanisms underpinning the anti‐atherosclerosis effects of EECP are gradually being revealed. (1) Increased shear stress: EECP increases vascular shear stress by 30‐60 dyne/cm^2^, within the range that is both beneficial and harmless.^16^, ^17^, ^18^ (2) Improvement in endothelial function: EECP improves endothelium‐dependent vascular relaxation by increasing plasma levels of nitric oxide while decreasing the levels of endothelin‐1, alleviates disarray of endothelial cells caused by hypercholesterolemia and increases levels of telomeric repeat binding factor 2.^17^, ^18^, ^19^ (3) Inhibition of oxidative stress and inflammation: EECP treatment results in a reduction in tumor necrosis factor‐alpha and monocyte chemotactic protein‐1 levels, improvement in hypercholesterolemia‐induced overexpression of p38 mitogen‐activated protein kinase, nuclear factor‐kappa B, vascular cell adhesion molecule‐1, etc.,^6^ all of which inhibits the progression of atherosclerosis. (4) Vasculogenesis and angiogenesis.^20^

## 3 APPLICATIONS OF EECP FOR CARDIOVASCULAR DISEASES IN THE ELDERLY

### 3.1 Coronary artery disease

An abundance of clinical studies have consistently confirmed the efficacy and safety of EECP for the treatment of angina. The MUlti‐center STudy of Enhanced External Counter Pulsation (MUST‐EECP) is the first multi‐center, prospective, randomized controlled trial that studied the effect of EECP on stable angina patients, of whom over 70% had received percutaneous coronary intervention (PCI) or coronary bypass grafting (CABG), 51% had a history of myocardial infarction (MI), 70% reported Canadian Cardiovascular Society (CCS) grade II or III symptomatic angina, 65% had multi‐vessel coronary artery disease. The results of this trial showed a significant improvement in exercise tolerance, time to 1 mm depression in ST segment, frequency in angina attacks and use of nitroglycerin after EECP treatment. Symptomatic improvement was sustained over one year in 70% of patients.^21^, ^22^ The Research on Enhanced external Counterpulsation therapy in Coronary artery disease (RECC) study found that EECP on top of optimal medical therapy improves myocardial ischemia and prognosis in patients with stable angina.^11^ In addition, EECP has been shown to induce collateral formation in the coronaries, thus it is expected to be useful for restenosis prevention in post‐PCI patients. The International EECP Patient Registry (IEPR) enrolled over 10 000 coronary artery disease patients from over 100 medical centers around the world. Outcomes of interest include CCS grading of angina symptoms, cardiovascular mortality, MI or re‐infarction, revascularization rate, and so on. Data from IEPR suggested that even one session of EECP treatment would bring immediate improvement in angina symptoms and quality of life, and the therapeutic benefit of EECP was sustained up to six months, one year, two years or even three years.^23^, ^24^ Despite the fact that most subjects in the above studies had revascularization procedures, and that about half had prior history of MI, and that a significant portion had cardiac dysfunction and/or diabetes, EECP was associated with clinical benefit among these patients.

Recently, increasing attention has been paid to non‐obstructive coronary artery diseases. EECP, with its positive effects on coronary perfusion and flow, as well as endothelial function, is believed to bring considerable benefits to these patients. Luo et al.^25^ reported that EECP treatment improved diastolic peak flow velocity and coronary flow reserve, along with relief in angina symptoms, in patients with angiographically confirmed coronary slow flow. These changes were associated with changes in flow‐mediated endothelial relaxation and levels of high‐sensitivity C‐reactive proteins. Tartaglia et al.^26^ assessed the effect of EECP on coronary microcirculation and discovered that 92% of patients showed improvement in myocardial perfusion scanning and exercise tolerance. Masuda et al.^27^ performed EECP treatment on aggravating angina patients and found that patients had better perfusion scanning on ischemic territories, better coronary flow reserve and better exercise tolerance.

### 3.2 Heart failure

Ischemic heart disease is the primary etiology of heart failure in the elderly population. Current evidence suggests that EECP on top of standard medical therapy improves quality of life and rehospitalization rates among patients with NYHA grade II to III stable heart failure. The Prospective Evaluation of EECP in Congestive Heart failure (PEECH) trial is a multi‐center prospective randomized controlled trial enrolling patients with symptomatic congestive heart failure (NYHA grade II to III, LVEF <35%). Its results showed that EECP improved exercise tolerance, cardiac function and quality of life, but not maximal oxygen intake at six months of follow‐up. Subgroup analysis further showed that among patients >65 years of age, maximal oxygen intake was increased after EECP treatment, indicating a greater extent of benefit for the elderly patients.^1^ Beck et al.^2^ reported that EECP improved vascular endothelial dysfunction, peripheral vascular resistance, myocardial perfusion, peripheral vascular function and exercise tolerance among patients with coronary artery disease and left ventricular dysfunction. Beck et al.^3^ also confirmed that EECP decreased left ventricular energy demand and myocardial oxygen demand as well as increased coronary perfusion and endocardial perfusion among patients with stable angina and heart failure.

In summary, the aforementioned MUST‐EECP, RECC, PEECH and IEPR studies had enrolled subjects aged 30 to 81 years (average 62), which means these results are applicable to the elderly population.^1^, ^22^, ^23^, ^24^ In the subgroup analysis of the PEECH trial, patients >65 years of age showed greater improvement in exercise tolerance.^1^ In the IEPR‐1 study, 8% of the subjects were >80 years old, who had similar benefits from EECP in terms of angina relief, increase in LVEF and so on.^23^, ^24^ One study conducted in China included coronary artery disease patients who were aged 80 or over and found that EECP could be relatively safe and used effectively among this population.^28^ Coronary artery disease in the elderly often present with diffuse lesions and chronic total occlusions. Thus elderly patients with coronary artery disease may often be deferred when contraindicated, or benefit less when occasionally indicated, from revascularization procedures. Theoretically, the presence of multiple comorbidities and the relatively low level of exercise tolerance in the elderly population renders EECP a more feasible option for these patients. Nevertheless, common features of frailty – myopenia, osteoporosis, osteoarthritis, etc. – in the elderly might result in early termination of EECP treatment due to skin damage or/and back pain. Clinical experience suggests that rigorous screening, standardized operating and close monitoring would ensure that 80% of elderly patients complete at least one session of EECP treatment. When managed appropriately, EECP can be a relatively low‐risk measure of non‐invasive therapy for elderly patients.

Recommendations for the application of EECP in cardiovascular diseases in the elderly include the following. (1) Coronary artery disease: a standard protocol of EECP is recommended for patients with angina, post‐MI, post‐PCI, post‐CABG, non‐obstructive coronary artery disease (especially coronary slow flow); (2) Heart failure: a standard protocol of EECP is recommended for chronic stable heart failure of ischemic origin (NYHA grade II to III).

## 4 CEREBROVASCULAR DISEASES OR OTHER NEUROLOGICAL DISEASES

### 4.1 Ischemic cerebrovascular diseases

Ischemic cerebrovascular diseases are common among the elderly, with relatively high mortality and morbidity. As research has shown that EECP facilitated cerebral blood flow regulation, enhanced collateralization of ischemic zone in the brain, and modulated levels of various cytokines, EECP can be considered as a helpful supplement in the rehabilitation in the acute phase of ischemic stroke.^29^ In a randomized cross‐over controlled trial, Han et al.^4^ reported that EECP can be administered early and in a safe manner to patients with acute ischemic stroke due to large vessel lesions. Lin et al.^30^ found that for patients with ischemic stroke receiving EECP treatment, average arterial pressure and therefore blood flow velocity in the middle cerebral artery was significantly increased, compared with only slight changes in the average arterial pressure and no change in cerebral blood flow in healthy controls, and they speculated that it was associated with cerebral autoregulation in control subjects. Xiong et al.^31^ studied the long‐term effect of cerebral blood flow and average arterial pressure during EECP treatment for acute ischemic stroke patients, and found that EECP increased cerebral blood flow up to three weeks, which gradually returned to baseline after one month, suggesting that EECP ought to be indicated within three weeks of onset. In the meantime, they also found that counterpulsation pressure of 150 mm Hg (0.020 MPa) had maximal effect on cerebral blood flow enhancement, and that total treatment sessions >10 hours were associated with improved outcome.^32^, ^33^ Therefore, currently, for patients with ischemic stroke, a total of >10 hours of EECP treatment with pressure at 150 mmHg is recommended. In addition, these authors found that EECP significantly reduces variability in blood pressure and heart rate,^34^, ^35^ which indicated that EECP improved autonomic dysfunction in patients with ischemic stroke. There were few studies of EECP on the posterior circulation. Werner et al.^36^ reported a 12% increase in blood flow to the vertebral artery during EECP treatment. Scholars in China also reported the beneficial effect of EECP on transient ischemic attack (TIA) patients due to vertebral‐basilar artery lesions.^37^

### 4.2 Neurodegenerative diseases

Incidence of neurodegenerative diseases is increasing. Presently in China, of those aged 65 years and older, about 1.7% have Parkinson's disease and 3%‐7% have Alzheimer's disease. Without any definitive cure currently, EECP therapy, a safe, non‐invasive treatment technique was attempted in these diseases. Zhou Qi et al.^38^ observed that among 33 patients with Parkinson's disease who received EECP treatment, 87.9% had symptomatic improvement as well as a reduction in Webster scores, probably due to increased cerebral blood flow, which in turn improved various neurotransmitters and receptor function in nigra dopaminergic neurons and brain stem. Scholars from China explored the effect of EECP on Alzheimer's disease and found that EECP treatment was associated with increased level of superoxide dismutase activity, somatostatin immunologic reaction SL1 and dynorphin AL‐13 levels in the blood and cerebrospinal fluids,^39^ indicating improved blood flow as well as a series of biochemical changes due to pulsatility impact.

### 4.3 Sleep disorder

Sleep disorder is common among the elderly, with incidence among the elderly aged 65 and over as high as 20%‐50%, manifested as sleep onset insomnia, early morning awakening and sleep pattern changes. Long‐term sleep disorder affects daily activities for elderly patients and is associated with the development of various neurological and psychiatric diseases. EECP improves cerebral blood flow, cellular oxygen and nutrition, as well as modulating related neurotransmitters, all of which improves insomnia symptoms. Multiple studies suggest that EECP is beneficial for insomnia related to coronary artery disease, hypertension, ischemic stroke and neurosis.^40^, ^41^ However, the dose‐effect relationship of EECP and insomnia improvement remains to be elucidated.

Recommendations for the application of EECP for neurological diseases in the elderly are as follows: (1) a standard protocol of EECP is indicated for patients with ischemic stroke who are in stabilized conditions (including blood pressure) in the acute phase, or for those in the subacute or chronic phases; (2) EECP can be considered in patients with TIA, chronic cerebral ischemia, especially with cerebral arterial stenoses; (3) EECP can be considered for patients with Parkinson's disease, Alzheimer's disease or other neurodegenerative diseases; (4) EECP can be considered in elderly patients with sleep disorders.

## 5 OTHER DISEASES IN THE ELDERLY

### 5.1 Type 2 diabetes with or without complications

A randomized controlled trial enrolling type 2 diabetes patients showed that a reduction in fasting glucose level, two‐hour post‐prandial glucose level and glycosylated hemoglobin level compared with baseline from EECP treatment at 48 hours and two weeks, and the effect on glycosylated hemoglobin reduction persisted to at least three months.^5^ These authors further investigated the glucose‐lowering effect of EECP and found that 48 hours after EECP treatment, insulin resistance, insulin sensitivity index, late glycosylated terminal products and receptors, oxidative stress and inflammation were all improved, the effects of which were present as long as six months.^5^, ^6^ In another study for patients with impaired glucose tolerance, EECP treatment resulted in decreased levels of tumor necrosis factor‐alpha and C‐reactive protein, and improvement in glucose intolerance, which was associated with improvement in alleviation of inflammation.^42^

Enhanced external counterpulsation has been shown not only to have glucose‐lowering effect, but also seemed to be beneficial for diabetic retinopathy, diabetic nephropathy and other chronic complications of diabetes. Compared with traditional therapy or laser photocoagulation, EECP treatment improved ophthalmic artery hemodynamics, fundal lesions and vision compared with traditional therapy.^43^ Scholars in China conducted many randomized controlled trials to investigate the effect of EECP on diabetic nephropathy with promising results. Compared with pharmacotherapy alone, EECP on top of pharmacotherapy reduces 24‐hour urine albumin, microalbumin, and urine and blood levels of beta‐microglobulin, which corresponded to a certain level of renal function preservation.^44^, ^45^ In addition, EECP has also been reported to yield favorable effects on diabetic foot, peripheral neuropathy and peripheral vascular diseases. Randomized controlled trials with a larger sample are warranted to further verify these benefits.

### 5.2 Ischemic eye diseases

Ischemic eye diseases in the elderly include central retinal artery embolism, ischemic optic neuropathy, ischemic optic atrophy, etc. Studies in China revealed that EECP can be beneficial in these conditions. A retrospective analysis of patients with ischemic eye diseases and carotid artery stenosis showed that patients had significant recovery of vision, sight and optic dynamics when managed with pharmacotherapy combined with EECP and that the earlier the treatment was administered, the better.^46^ Another study enrolling patients with non‐arteritic anterior ischemic optic neuropathy revealed that EECP increased average flow velocity, peak systolic and end‐diastolic flow of both ophthalmic arteries and central retinal artery, accompanied by improvement in vision and hemodynamic parameters, in which the former was positively correlated with the latter, with greater improvement in the affected side compared with the intact side.^7^

### 5.3 Sudden hearing loss

Enhanced external counterpulsation has been shown to be beneficial for sudden hearing loss. In a study done by Offergeld et al.,^8^ for patients with acute persistent hearing loss and/or tinnitus, EECP treatment resulted in a 19% increase in carotid flow and an 11% increase in vertebral flow, and 47% of patients reported an improvement in tinnitus symptoms, 28% an improvement in hearing, which was maintained at one year of audiometric follow‐up after EECP treatment.

### 5.4 Erectile dysfunction

Not only does erectile dysfunction share common risk factors with cardiovascular diseases, but both conditions can influence each other in many ways. Studies found that EECP treatment improves erectile dysfunction with or without refractory angina.^9^ Froschermaier et al.^47^ reported a significant increase in peak systolic flow of corpus cavernosum artery and a subsequent improvement in erectile function in these patients, which was sustained at 6.70 ± 4.37 months. Lawson et al.^48^ pointed out that EECP was beneficial for erectile dysfunction in patients with refractory angina in erectile function per se as well as sexual satisfaction, but not in orgasm and libido. Currently, erectile dysfunction is primarily managed with phosphodiesterase type 5 (PDE‐5) inhibitor. However, PDE‐5 inhibitors are often contraindicated in many coronary artery disease patients because of concomitant use of nitrates. EECP with its benefits both in cardiac and erectile function can be an immensely useful alternative for these patients.

### 5.5 Psychological and psychiatric diseases

Evidence from clinical trials indicates that EECP improves psychological and psychiatric symptoms, along with social function and work capacity. MUST‐EECP showed that EECP improves daily activity, work, body pain, confidence, stamina, social activities, anxiety and depression among patients with angina.^22^ Fricchione et al.^49^ reported a significant improvement in subjective perception, quality of life, and general well‐being among patients with refractory angina who received EECP, even though myocardial perfusion was not improved. They also discovered that EECP improved depression, anxiety and somatic symptoms, but not anger and hostility, and that these improvements were more prominent among patients with objective evidence of improvements in myocardial ischemia.^10^

Recommendations of the application of EECP for other diseases in the elderly are as follows: (1) a standard protocol of EECP treatment is recommended for patients with various ischemic diseases and type 2 diabetes; (2) EECP can be considered for type 2 diabetes patients for better glycemic control who have managed lifestyle and pharmacotherapy; (3) EECP can be considered in patients with diabetic retinopathy and nephropathy; (4) EECP is recommended as early as possible for patients with central retinal artery embolism, ischemic ophthalmic nephropathy and atrophy, and for those in the chronic phase EECP can also be considered; (5) EECP is recommended as early as possible for patients with sudden hearing loss, and for those in the chronic phase EECP can also be considered; (6) a standard protocol of EECP is recommended for coronary artery disease patients with erectile dysfunction; (7) EECP can be considered for patients with erectile dysfunction where traditional treatments have failed; (8) EECP is recommended for patients with ischemic diseases who have anxiety or depression.

## 6 INDICATIONS AND CONTRAINDICATIONS OF EECP

### 6.1 Indications

- Cardiovascular diseases. (1) coronary artery diseases: angina, post‐MI, post‐PCI, post‐CABG, non‐obstructive coronary artery diseases; (2) chronic stable heart failure (of ischemic origin, NYHA grade II to III).
- Neurological diseases. (1) ischemic stroke; (2) transient ischemic attack; (3) Parkinson's disease; (4) Alzheimer's disease; (5) sleep disorder.
- Other diseases in the elderly: (1) ischemic diseases with type 2 diabetes; (2) type 2 diabetes with suboptimal glucose control after lifestyle modification and pharmacotherapy; (3) diabetic retinopathy and nephropathy; (4) central retinal artery embolism, ischemic optic neuropathy and ischemic optic atrophy; (5) sudden hearing loss; (6) coronary artery disease with erectile dysfunction; (7) erectile dysfunction refractory to traditional treatments; (8) ischemic diseases with anxiety or depression.

### 6.2 Contraindications

- Deep venous thrombosis or active thrombotic phlebitis in the lower extremities
- Moderate‐to‐severe valvular lesions, especially aortic regurgitation
- Moderate‐to‐severe pulmonary hypertension (mean pulmonary artery pressure >50 mm Hg)
- Aortic or cerebral aneurysm
- Uncontrolled hypertension (>180/110 mm Hg)
- Decompensated heart failure
- Arrhythmias that might possibly interfere with ECG‐gated function
- Bleeding disorder or diathesis
- Active infection in the lower extremities.

## 7 SAFETY ASSESSMENT, RISK MANAGEMENT, AND OPERATIONAL ISSUES

### 7.1 Safety assessment

Although EECP is a relatively safe and well‐developed treatment, a safety assessment to rule out contraindications and lower treatment‐related risks is necessary for high‐risk and elderly patients before administration.

### 7.2 Basic assessment

Basic assessment includes an assessment of the general condition of the patient, the presence or absence of comorbidities and complications, complete blood count, coagulation panel, lipid profile, glucose panel, liver and kidney function tests, routine ECG, echocardiography, lower extremities ultrasound and so on.

### 7.3 Targeted assessment

For some severe cases, targeted assessment is recommended, which may include Holter ECG, ambulatory blood pressure monitoring, non‐invasive hemodynamic examinations, and so on.

### 7.4 Risk management

- Patients with hypertension should have their blood pressure controlled at <150/90 mm Hg; patients with acute ischemic stroke can safely receive EECP when their blood pressure is <180/100 mm Hg.
- Patients with tachycardia should have their heart rate controlled at <100 bpm.
- Atrial fibrillation may result in irregular inflation/deflation cycle due to irregular heart rate, reducing comfortability level during treatment. A ventricular rate of 50‐90 bpm is recommended for these patients. EECP should not be administered to atrial fibrillation patients with atrial thrombus.
- Patients with heart failure should be monitored for their heart rate, oxygen saturation, the presence of rales and respiratory rate, and occasionally non‐invasive hemodynamic monitoring. Acute decompensated heart failure patients with volume overload should have their conditions stabilized before receiving EECP.
- Ventricular aneurysm is not an absolute contraindication for EECP, but caution should be used with patients with ventricular aneurysm that is large, thin walled, with left ventricular dysfunction, or ventricular thrombus.
- Obstructive atherosclerotic diseases in the lower extremities, including severe stenosis or occlusion of the arteries, may benefit from EECP,^50^ but treatment should start with low counterpulsation pressure and short time span, and gradually increase in response to patient tolerance. Close monitoring is mandated, and treatment should be immediately discontinued in the presence of severe adverse reaction. Pneumatic cuffs should not be placed around arterial segments that have been treated with stent implantation.
- Consultation with orthopedic surgeons and rehabilitation physicians for patients with severe osteoporosis, a history of hip replacement or femoral surgeries before EECP treatment; due to passive movements of body during treatment, EECP should be cautiously administered to patients with lumbar herniated disk.
- Elderly patients on warfarin should have their PT‐INR controlled at <2.5 before receiving EECP.
- Patients with a permanent pacemaker with rate‐response function may develop inappropriate tachycardia due to body movements during treatment wrongly sensed as input. Simply turning off this function during treatment is sufficient. No extra interrogation is needed for patients with an implantable cardioverter‐defibrillator.
- Elderly patients with diabetes may be prone to skin damage during EECP treatment, which can be prevented by elasticated stockings.
- EECP may result in an increase in urinary frequency and urgency, which may be more prominent among the elderly. Asking patients to empty their bladders before treatment is advisable. When the patient expresses a need to go to bathroom, promptly stop treatment to avoid a rise in heart rate and blood pressure, which negatively affects treatment effect and compliance. A diaper is occasionally needed for some patients.

### 7.5 Operational issues

- Positioning of ECG electrodes: ECG electrodes should be placed in the precordium for maximal R wave amplitude. Interference can be avoided by cleaning and drying the skin (e.g. with an alcohol swab) before placing electrodes to ensure close attachment.^11^
- The pneumatic cuffs should be wrapped tightly, especially for the proximal side, and checked regularly during treatment to see whether they have become loosened over time.
- Inflation pressure: choose minimal pressure with optimal counterpulsation wave according to the condition, body weight and tolerance of the patient.
- Inflation/deflation time: normally, begin inflation at T wave and deflation at the beginning of P wave. Inflation/deflation time can be adjusted to ensure maximal counterpulsation wave amplitude, D/S ratio and DP/SP ratio.
- For coronary artery disease patients, optimal hemodynamic effect is achieved at D/S > 1.2 and DP/SP 1.5–2.0. Try to use appropriately sized cuffs, ensure adequate wrapping, and adjust inflation pressure and inflation/deflation time to achieve optimal ratios.
- Monitor oxygen saturation during treatment, and promptly stop when oxygen saturation gradually decreases to <90%. Look for underlying causes and treat accordingly.
- Inner trigger mode should not be used.

## 8 TREATMENT PROTOCOL, MONITORING OF EFFICACY DURING AND AFTER TREATMENT

### 8.1 Treatment protocol

Optimal treatment efficacy relies on an individualized treatment protocol tailored to different conditions and clinical scenarios.

#### 8.1.1 Treatment parameters

Start with low inflation pressure and gradually increase to target range over three to five cycles. Target inflation pressure varies according to different conditions: 0.020‐0.035 MPa brings maximal alleviation of myocardial ischemic in elderly angina patients; 0.020 MPa brings maximal cerebral blood flow for patients with ischemic cerebrovascular diseases.^32^ In the meantime, skin fat and muscular mass should be taken into consideration when setting inflation pressure: higher for the relatively obese patients, and lower for the relatively lean patients. Aim at D/S > 1.2 and DP/SP 1.5‐2.0 for optimal effect by adjusting inflation pressure and inflation/deflation time. Due to factors like atherosclerotic vascular disease in the lower extremities, multi‐vessel disease, or vessel obstructions, optimal ratios may not be achieved in the elderly. However, clinical practice confirms that EECP can still be beneficial despite suboptimal ratios. Reasons for this phenomenon may be other mechanisms of benefit involved besides immediate hemodynamics, e.g. shear stress enhancement, improvement in endothelial function, promotion of angiogenesis, and so on.

#### 8.1.2 Treatment sessions

Standard protocol of EECP for ischemic cardio‐cerebrovascular diseases is: one hour per day, given in one or two sessions. Treatment time can be shortened for less tolerant patients. The two‐hour‐per‐day measure is also proposed but its effect remains to be investigated. A total of 36 hours over six weeks (six days per week) or a total of 35 hours over seven weeks (five days per week) are standard protocol. A short version of 10‐ to 12‐hour sessions has been shown to be beneficial for angina relief. Total hours of treatment session can be adjusted according to patients’ condition and response. An extended session for another 10 to 12 hours is recommended for severe coronary artery disease patients. The mid‐ to long‐term effect of EECP is closely related to total hours of treatment. Two standard protocols of EECP per year on a regular basis are recommended for most ischemic cardio‐cerebrovascular disease patients. An additional one or two standard protocols are advisable for triple‐vessel disease and chronic heart failure patients. Upon completion of the standard protocol, two to three hours per week of maintenance treatment is a favorable option also.

### 8.2 Monitoring of efficacy during treatment

It is crucial to monitor treatment efficacy during EECP sessions. Parameters are adjusted accordingly to ensure optimal benefit. Different conditions require different data points to be monitored. Indices for immediate hemodynamic effects are inflation/deflation time, inflation pressure, ECG, blood pressure, D/S ratio and DP/SP ratio, and in some cases, stroke volume, cardiac output, myocardial contractility, cardiac preload, peripheral vascular resistance. Data should be stored and retrievable at a later time. For patients with heart failure, non‐invasive hemodynamic monitoring is recommended.

### 8.3 Monitoring of efficacy after treatment

Hemodynamic indices can be used to evaluate EECP treatment efficacy in terms of its immediate hemodynamic effects, but they are not the primary efficacy parameters. Symptomatic improvement, as well as mid‐ to long‐term effect should be priorities. Vascular function testing such as atherosclerotic burden, vascular stiffness, endothelial function, along with echocardiography, non‐invasive cardiac function, treadmill test, six‐minute walk test, cardiopulmonary assessment, neurological evaluation, quality of life assessment, general geriatric assessment and so on are important measures to evaluate the mid‐ to long‐term effects of EECP treatment.

## CONFLICTS OF INTEREST

Nothing to disclose.

## Appendix A

**Authors:** Shen Lin, Leng Xiuyu, Xiong Li, Zhao Shaohua

**Supervisors:** Hu Dayi, Zhang Yun

**Expert Committee Expert committees are in alphabetical order:** Bu peili (Department of cardiology, Qilu Hospital of Shandong University), Chen Yuguo (Emergency Department, Qilu Hospital of Shandong University), Cheng Mei (Department of Geriatrics, Qilu Hospital of Shandong University), Dong Yugang (Department of Cardiovascular Medicine, The First Affiliated Hospital of Sun Yat‐sen University), Duan Chunbo (National Geriatric Center, Editorial Department of Peking University), Fan Zhiqing (Department of Cardiology, Daqing oilfield General Hospital), Fang Ningyuan (Department of Geriatrics, Tongji Hospital, The Affiliated Hospital of Shanghai Jiaotong University School of Medicine), Gao Haiqing (Department of Geriatrics, Qilu Hospital of Shandong University), Gao Wei (Department of Cardiology, Peking University Third Hospital), Gao Wenxue (Inner Mongolia People's Hospital), Hong Huashan (Fujian Medical University Union Hospital), Hu Dayi (Institute of Cardiovascular Disease, Peking University People's Hospital), Ji Xiaoping (Department of Cardiology, Qilu Hospital of Shandong University), Leng Xiuyu (Department of Cardiovascular Rehabilitation, The First Affiliated Hospital of Sun Yat‐sen University), Li Xi (Department of Geriatrics, The Second Affiliated Hospital of Xi'an Jiaotong University), Li Xiaoying (Department of Geriatric Cardiology, Chinese PLA General Hospital), Lin Zhanyi (Department of Geriatrics, Guangdong Provincial People's Hospital), Liu Weijing (Department of Cardiology, Shanghai Tenth People's Hospital), Lu Feng (Department of Heart Disease, The Affiliated Hospital of Shandong University), Lu Qinghua (Department of Cardiology, The Second Hospital of Shandong University), Meng Xiaoping (Cardiac Rehabilitation Center, The Affiliated Hospital of Changchun University of Chinese Medicine), Shenlin (Department of Geriatrics, Qilu Hospital of Shandong University), Sun Yanling (Department of Cardiology, Luoyang Hospital of TCM), Wang Chaohui (Department of Geriatrics, Union Hospital Affiliated to Tongji Medical College of Huazhong University of Science and Technology), Wang Jianye (National Center of Geriatrics, Peking Hospital), Wang Xiaoming (Department of Geriatrics, Jingxi Hospital, Air Force Medical University of PLA), Wei Fengtao (Department of Cardiology, The Second Hospital of Shandong University), Wu Guifu (The Eighth Affiliated Hospital of Sun Yet‐Sen University), Wu Wei (Department of Neurology, Qilu Hospital of Shandong University), Wu Yongjian (Coronary Heart Disease Center, Fuwai Hospital of CAMS&PUMC), Xiong Li (Department of Neurology of Medicine and Therapeutic, The Chinese University of HongKong), Xu Danping (Department of Cardiovascular, Guangdong Provincial Chinese Medicine Hospital), Xu Feng (Department of Emergency, Qilu Hospital of Shandong University), Xu Yawei (Department of Cardiology, Shanghai Tenth People's Hospital, Yang Ruiying (General Hospital of Ningxia Medical University), Yang Tianlun (Department of cardiovascular Medicine, Xiangya Hospital, Central South University), Yang Yunmei (Department of Geriatrics, The First Affiliated Hospital of Zhejiang University), You Bei'an (Department of Cardiology, Qilu Hospital of Shandong University), Yu Pulin (National Center of Geriatrics,Institute of Geriatrics of Peking Universtity), Yuan Haitao (Departement of Cardiology, Shandong Provincial Hospital), Yue Shouwei (Department of Rehabilitation, Qilu Hospital of Shandong University), Zhang Cuntai (Department of Geriatrics, Tongji Hospital, Tongji Medical College Huazhong University of Science & Technology), Zhang Hui (Department of Cardiovascular Medicine, The Second Affiliated Hospital of Zhengzhou University), Zhang Huijie (Department of Functional Testing and Rehabilitation, Shenzhen Branch of Fuwai Hospital, Chinese Academy of Medical Sciences), Zhang Yun (Department of Cardiology, Qilu Hospital of Shandong University), Zhao Mingming (Cardiopulmonary Rehabilitation Center, The Third People's Hospital of Guangxi Zhuang Autonomous Region), Zhao Shaohua (Department of Geriatrics, Qilu Hospital of Shandong University).
